# BRAF Detection in FNAC Combined with Semi-Quantitative ^99m^Tc-MIBI Technique and AI Model, an Economic and Efficient Predicting Tool for Malignancy in Thyroid Nodules

**DOI:** 10.3390/diagnostics14131398

**Published:** 2024-06-30

**Authors:** Laura Teodoriu, Maria-Christina Ungureanu, Mioara Matei, Irena Grierosu, Alexandra Iuliana Saviuc, Jalloul Wael, Iuliu Ivanov, Loredana Dragos, Radu Danila, Velicescu Cristian, Mihai-Andrei Costandache, Adrian Iftene, Cristina Preda, Cipriana Stefanescu

**Affiliations:** 1 Endocrinology Department, “Grigore T. Popa” University of Medicine and Pharmacy, 700111 Iasi, Romania; laurateodoriu@gmail.com (L.T.);; 2 Preventive Medicine and Interdisciplinarity Department, “Grigore T. Popa” University of Medicine and Pharmacy, 700111 Iasi, Romania; 3 Biophysics and Medical Physics—Nuclear Medicine Department, “Grigore T. Popa” University of Medicine and Pharmacy, 700111 Iasi, Romania; 4 Center of Fundamental Research and Experimental Development in Translational Medicine, Regional Institute of Oncology, 700483 Iasi, Romania; 5 Department of Surgery, Faculty of Medicine, “Grigore T. Popa” University of Medicine and Pharmacy, 700111 Iasi, Romania; 6 Faculty of Computer Science, “Alexandru Ioan Cuza” University, 700506 Iasi, Romania

**Keywords:** BRAF in FNAC, ^99m^Tc-MIBI wash-out, thyroid carcinoma, AI

## Abstract

**Simple Summary:**

Accurate preoperative diagnosis of the nature of thyroid nodules remains a challenge and generates a constant demand for improving the diagnostic tools. The aim of the study was to create a more efficient and yet economic diagnostic tool by combining multiple diagnostic techniques prone to daily use. In our research, we combined ultrasound findings, BRAF mutations in fine needle aspiration cytology (FNAC) and functional imaging with a semi-quantitative technique: ^99m^Tc-methoxy-isobutyl-isonitrile ([^99m^Tc]MIBI) wash-out. Preoperative findings were correlated with a histological report from thyroidectomy, which wasable to assess a diagnostic pattern with the help of an artificial intelligence (AI) model for our small cohort.

**Abstract:**

Background: Technology allows us to predict a histopathological diagnosis, but the high costs prevent the large-scale use of these possibilities. The current liberal indication for surgery in benign thyroid conditions led to a rising frequency of incidental thyroid carcinoma, especially low-risk papillary micro-carcinomas. Methods: We selected a cohort of 148 patients with thyroid nodules by ultrasound characteristics and investigated them by fine needle aspiration cytology (FNAC)and prospective BRAF collection for 70 patients. Also, we selected 44 patients with thyroid nodules using semi-quantitative functional imaging with an oncological, ^99m^Tc-methoxy-isobutyl-isonitrile (^99m^Tc-MIBI) radiotracer. Results: Following a correlation with final histopathological reports in patients who underwent thyroidectomy, we introduced the results in a machine learning program (AI) in order to obtain a pattern. For semi-quantitative functional visual pattern imaging, we found a sensitivity of 33%, a specificity of 66.67%, an accuracy of 60% and a negative predicting value (NPV) of 88.6%. For the wash-out index (WOind), we found a sensitivity of 57.14%, a specificity of 50%, an accuracy of 70% and an NPV of 90.06%.The results of BRAF in FNAC included 87.50% sensitivity, 75.00% specificity, 83.33% accuracy, 75.00% NPV and 87.50% PPV. The prevalence of malignancy in our small cohort was 11.4%. Conclusions: We intend to continue combining preoperative investigations such as molecular detection in FNAC, ^99m^Tc-MIBI scanning and AI training with the obtained results on a larger cohort. The combination of these investigations may generate an efficient and cost-effective diagnostic tool, but confirmation of the results on a larger scale is necessary.

## 1. Introduction

Thyroid nodules are very common, and their prevalence is rising from decade to decade. They are discovered either clinically from self-palpation by the patient, during a health checkup by a clinician, or incidentally during an imaging procedure such as ultrasonography (US), computed tomography (CT),or magnetic resonance imaging (MRI), or 18Ffluorodeoxyglucose (FDG) positron emission tomography (PET) for other indications. With the increased use of sensitive imaging techniques, thyroid nodules are being diagnosed incidentally with increasing frequency in the recent years [1].

Globally, in 2020, the age-standardized incidence rates of thyroid cancer were 10.1 per 100,000 women and 3.1 per 100,000 men, and age-standardized mortality rates were 0.5 per 100,000 women and 0.3 per 100,000 men as reported by Pizzato et al. [2].

The journey of discovering thyroid nodules and then diagnosing the thyroid cancer is complicated and needs a lot of energy and resources. The investigations usually required are thyroid US, FNAC, and functional imaging such as a thyroid scan with planar images or single-photon emission computerized tomography (SPECT). The exploration for thyroid nodules begins with US characteristics that can be determined using the Thyroid Imaging Reporting and Data System (TIRADS) calculator, which consists of nodular composition, echogenicity, shape, margins and echogenic foci [3]. Depending on the TIRADS (TR 3,4,5)calculated score, it will be necessary to perform FNAC, and the cytology result reported by The Bethesda System for Reporting Thyroid Cytopathology (TBSRTC) will establish the indication for surgery.

TBSRTC 2023 recommends the following six reporting category names: (I) non-diagnostic; (II) benign; (III) atypia of undetermined significance (AUS); (IV) follicular neoplasm; (V) suspicious for malignancy (SFM); and (VI)malignant. Atypia of undetermined significance (Bethesda III) has a risk of malignancy (ROM) of 22% (13–30%), and the current recommendations are repeat FNA, molecular testing, diagnostic lobectomy or surveillance. AUS with nuclear atypia (AUS-NA) has a significantly higher ROM compared with AUS associated with other patterns: architectural atypia, oncocytic atypia, and lymphocytic atypia. Follicular neoplasm (Bethesda IV) has a risk of malignancy of 30% (23–34%), and the recommendations are molecular testing and diagnostic lobectomy [4]. There is a notable upward trend in terms of recommending molecular determinations in the case of Bethesda III and IV cytopathological results.

The risk of malignancy is much higher in the case of nuclear atypia such as nuclear grooves, chromatin margination, nuclear molding, nuclear contour irregularity and nuclear overlapping on FNAC [5]. Also, BRAF^V600E^ had the highest rate of positivity in AUS-NA, about 40%, compared with 30.8% AUS with anoncocytic pattern and 6.7% for AUS with other patterns [6].

As reported in the systematic review performed by Goldner and colleagues [7], the BRAF^V600E^ mutation alone has the highest PPV amounting to 98%, whereas other common alterations have lower PPV, such as the fusion PAX8/PPARG (55%), HRAS^Q61R^ (45%), BRAF^K601E^ (42%), and NRAS^Q61R^ (38%) in detecting thyroid cancer [8]. BRAF^V600E^ was more associated with papillary thyroid carcinoma (PTC: 53.0%) than follicular thyroid carcinoma (FTC: 10.0%) [9].

As the costs of the molecular tests decreases, it tends to become the first-line management of indeterminate solitary thyroid nodules. Other molecular tests are in development and are likely to be made available at lower costs. For indeterminate thyroid nodules, the results of Dharampal et al. showed that in regard to costs, molecular testing is a superior strategy compared to standard management in decreasing the number of unnecessary surgeries. The incremental cost is $4234.22CAD = 3135.04 USD = 2985.60 EUR per unnecessary surgery avoided [10].

In Bethesda I nodules, BRAF^V600E^ showed similar sensitivity to TIRADS evaluation. BRAF^V600E^ had higher specificity and lower accuracy compared to TIRADS (99.4% vs. 88.9%, *p* < 0.001; 94.6% vs. 96.6%, *p* < 0.01) as written by Wu et al. [11]. In Bethesda III/V nodules, the sensitivity, specificity and accuracy of BRAF^V600E^ were similar to those of TIRADS [11].

Different techniques are used for obtaining thyroid smears, and diagnostic performance can be influenced by that. Rapid on-site evaluation (ROSE) by a pathologist or cytologist is improving the sample adequacy and decreasing the number of needle passes [12,13]. Rufail et al. demonstrated that the combined use of conventional smear and liquid-based preparation (LBP) resulted in a significant decrease in Bethesda IV rate and an increase in the benign rate [13,14].

Dell’Aquila et al. use this LBP technique extracting DNA from both LBC-stored, aspirated material and paraffin-embedded tissues. A correlation of BRAF^V600E^ and thyroid disease stage was detected, indicating a significant *p* value of 0.0009 for stages II and III. Researchers also found a higher correlation with the BRAF and TERT mutations in PTC with lymph node metastases (*p* = 0.0349) [15]. LBC samples were also used for determining p53 mutation, being evaluated by direct DNA sequencing of the thyroid, and the results showed that PTC cases with p53 expression had a more aggressive behavior in terms of multifocality, nodal metastases, and larger size (>1.5 cm) [16].

Machine learning is in continuous use in thyroid nodules diagnosis, being used to discriminate malignant nodules from benign ones. US characteristics and functional imaging are the main resources for AI diagnosis results.

AI seems to match experienced radiologists in US evaluation in terms of accuracy, as published in a recent review. Thus, it can be successfully used in computer-aided diagnosis (CAD), which is a solution that would be particularly helpful for less experienced physicians. There would be significant benefits for patients, including the reduction in unnecessary FNAC and unnecessary surgeries [17].

Of the 21048 diagnoses made by 12 radiologists, the ThyNet-assisted strategy reduced FNAC from 61.9% to 35.2% (*p* < 0.0001), while missed malignancy decreased from 18.9% to 17.0% (*p* < 0.0001). The combination of the ACR TIRADS classification with AI assistance improved the NPV and PPV, which has the potential to reduce the number of unnecessary FNAC. Of the 1754 nodules assessed, a median of 134 FNAC could have been avoided with the assistance of ThyNet due to the high probability of being malignant [18].

We propose, as a study line, the combination of thyroid US with BRAF^V600E^ from residual FNAC material and functional imaging using oncological ^99m^Tc-MIBI radiotracer wash-out of thyroid nodules, the aim being to develop a functional imagistic pattern for suspicious thyroid nodules and also to correlate presurgical molecular findings (BRAF mutation) with the final histopathological result. We propose also an AI model trained with US characteristic (hypoechogenicity, microcalcifications) and ^99m^Tc-MIBI wash-out results and visual pattern results for those patients who were surgically treated in order to develop a model that could apply to patients who have not been operated on but with the same US and ^99m^Tc-MIBI characteristics.

## 2. Materials and Methods

This is a prospective, non-interventional study that has been approved by the ethics committee of the “Gr. T. Popa” University of Medicine and Pharmacy and Regional Oncology Institute, Iasi. All participants provided written informed consent, and clinical procedures were conducted according to the Declaration of Helsinki.

**Figure 1:**
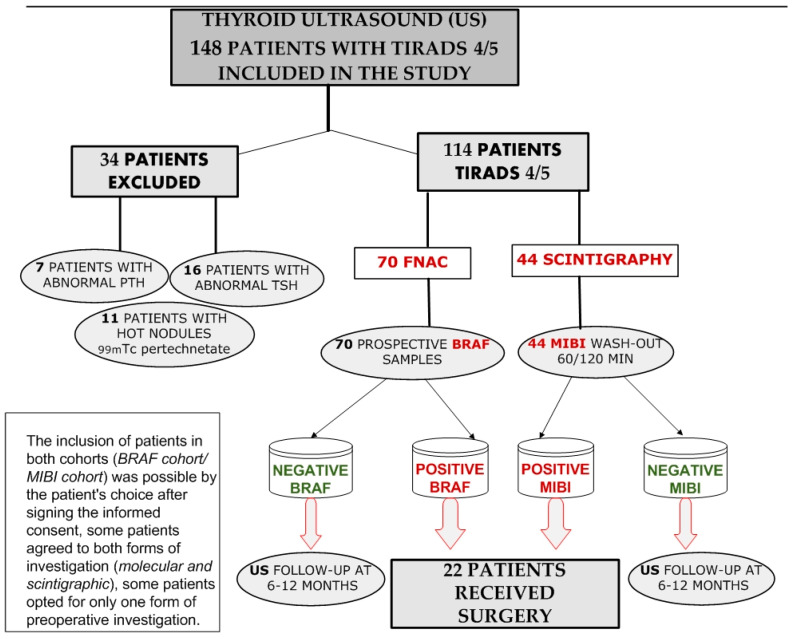
Our study algorithm. US—ultrasound, FNAC—fine needle aspiration cytology.

This study was carried out during October 2020–October 2022 (2 years). A number of 148 patients were included in this study; 44 patients were selected for ^99m^Tc-MIBI imaging and 70 patients were selected for BRAF molecular testing (Figure 1). The inclusion criteria for both cohorts (MIBI cohort and BRAF cohort) of the study were established according to the TIRADS calculation. We prospectively obtained 210 molecular results from BRAF cohort patients and 135 images of ^99m^Tc-MIBI for MIBI cohort patients with thyroid nodules over 1 cm in diameter with calculated TIRADS of high risk. The remaining patients were excluded due to modified hormonal values of the thyroid-stimulating hormone (TSH), parathormone (PTH) and also finding hot nodules results on ^99m^Tc pertechnetate scans.

### 2.1. Patients

The inclusion criteria are outlined below:

1. Age > 18 years;
2. Normal thyroid-stimulating hormone (TSH—normal values chemiluminescent: 0.48–4.17 mUI/L) and calcitonin levels (Calcitonin—normal values chemiluminescent: 0–5 pg/mL);
3. Thyroid nodules of at least 10 mm in maximum diameter, TIRADS risk class 4 (intermediate-risk) or 5 (high-risk) as determined by thyroid US.

The exclusion criteria are outlined below:

1. Abnormal TSH;
2. Hyperparathyroidism;
3. Age under 18 years old.

### 2.2. Fine Needle Aspiration Cytology (BRAF Cohort)

FNAC was performed under US guidance with a 22-gauge needle attached to a 10mL plastic syringe by an endocrinologist. Part of the aspirated fluid was expelled and smeared onto charged slides and then air-dried and processed for Giemsa staining. The material for the isolation of DNA was washed from the needle with 1 mL sterile 0.9% saline solution after the FNAC procedure. The puncture tissue was stored in a 1.5 mL Eppendorf tube at −20 °C. The remaining material was stored frozen for potential further molecular examination. DNA was isolated from the FNAC samples and extracted by the Genesig^®^ Primerdesign™ York House, School Lane, Chandler’s Ford Eastleigh, SO53 4DG, UK following the manufacturer’s protocol (BRAF^V600E^ mutation detection by quantitative allele specific amplification (quasa) BRAF^V600E^ c.1799T > A, BRAF^V600E^ c.1799_1800TG > AA, BRAF^V600K^ c.1798_1799GT > AA).

### 2.3. ^99m^Tc-MIBI Imaging (MIBI Cohort)

Thyroid scans were obtained by using a dual headed gammacamera equipped with low-energy, high-resolution parallel-hole collimators manufactured by SIEMENS (Station Name SYMBIA EVO EXCEL 1045, Regional Institute of Oncology, Iasi, Romania). Planar anterior images (ZOOM: 2; matrix 256 × 256; frame time: 250 × 10^3^ counts, energy peak: 140 ± 20 KeV). Early^99m^Tc-MIBI images were acquired 20 min (median) and late images were acquired 60 min (median) and 120 min after injection of 500–740 MBq of the radiopharmaceutical (RF) [19]. For visual analysis, early ^99m^Tc-MIBI scans were described according to the following criteria.

### 2.4. ^99m^Tc-MIBI Image Analyses

The image acquisition protocol began with the examination of ^99m^Tc pertechnetate, obtaining images 20 min after RF injection. Hypofunctional nodules were included in the ^99m^Tc-MIBI scanning study. We used the two days protocol of acquiring images: the first day ^99m^Tc pertechnetate, and after that, ^99m^Tc-MIBI scanning.

Visual analysis was in accordance with literature data and also with our ^99m^Tc-MIBI scan protocol [20,21,22,23] and included the evaluation of early and late planar images, and the ^99m^Tc-MIBI kinetics within thyroid nodules were also assessed. Additional semi-quantitative results (WOind) were available for each patient.

**Figure 2:**
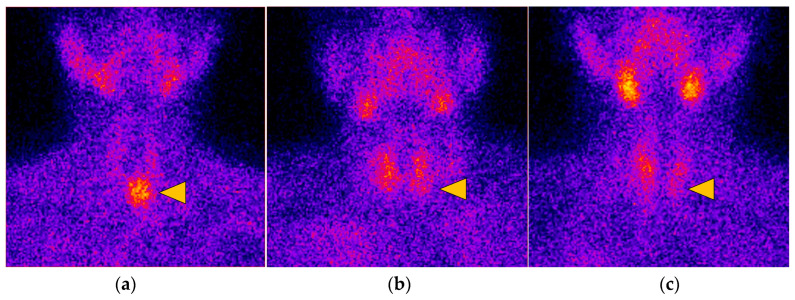
Thyroid nodule types represented by scans with varying ^99m^Tc-MIBI uptake compared to paranodular thyroid tissue: (**a**) Nodule (yellow triangle) with hyperintense RF uptake (uptake TN > uptake paranodular); (**b**) Nodule (yellow triangle) with isointense RF uptake (uptake TN = uptake paranodular); (**c**) Nodule (yellow triangle) with hypointense RF uptake (uptake TN < uptake in paranodular tissue).

(1) For both early and late planar images, the visual method described the ^99m^Tc-MIBI uptake in the thyroid nodule (TN) compared to the paranodular thyroid tissue as *hypointense*(uptake TN < uptake in paranodular tissue), *isointense* (uptake TN = uptake paranodular),and *hyperintense* (uptake TN > uptake paranodular) (Figure 2). A hypointense ^99m^Tc-MIBI uptake was defined as benign, isointense or hyperintense ^99m^Tc-MIBI uptake was considered suspicious for malignancy.
(2) The visual assessment of wash-out kinetics of ^99m^Tc-MIBI was classified as follows:
  (i) Visual pattern A: reduced uptake in the nodule in the early and late image;
  (ii) Visual pattern B: uptake in the nodule that decreases from early to late image;
  (iii) Visual pattern C: uptake in the nodule that remains unchanged or has further increased on the delayed image [20,21,22].

Pattern C was considered suspicious for malignancy, whereas pattern A and B both were considered indicative for a benign TN.

**Figure 3:**
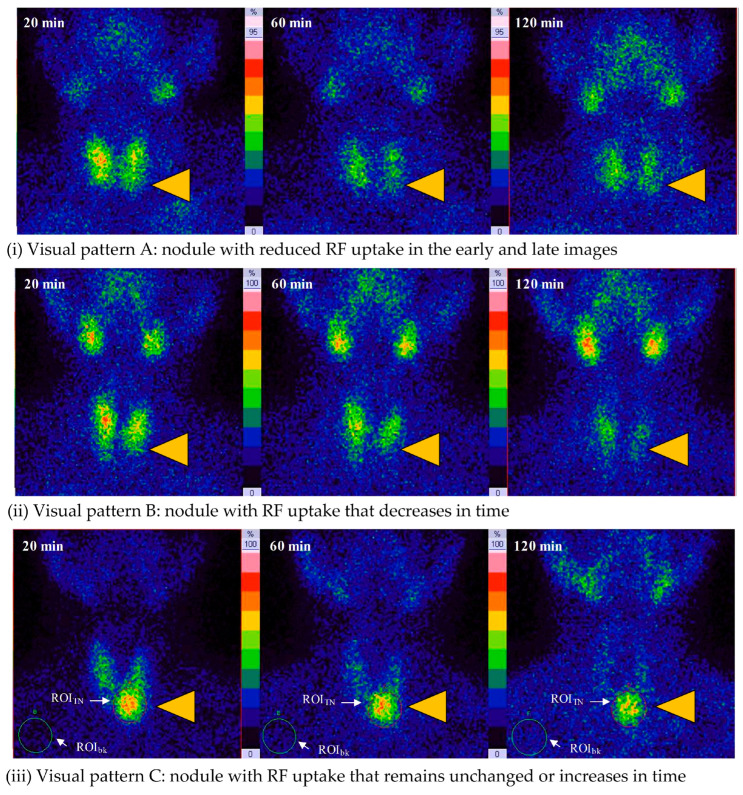
Visual patterns (TN are marked by yellow triangle) regarding the kinetics of ^99m^Tc-MIBI uptake in TNs.

The WOind quantifies the percentage ^99m^Tc-MIBI uptake reduction in a TN between the early and the late image. A region of interest (ROI) was drawn manually around the TN of interest on the early image (TN early) and then mirrored outside the thyroid gland for subtracting the background (Bk) activity (Bk on the early image), as seen in Figure 3(iii).

The early result (ER) was calculated: mean counts TN early—mean counts Bk early.

Subsequently, both ROIs were copied onto the late image (TN late, Bk late).

The late result (LR) was calculated: mean counts TN late—mean counts Bk late.

For calculating the wash-out index, the following formula was used according to Campenni et al.: WOind (%) = LR/ER × 100 − 100 [20,21,22].

### 2.5. Statistical Analysis

Sensitivity, specificity, PPV, NPV, and accuracy were calculated for each detection method and combined methods, considering histology as the gold standard. All data were processed using Microsoft Excel 2019.

### 2.6. AI Model

In order to construct our AI model, we employed a quite complex Python code along with the NumPy and TensorFlow-Keras libraries for interpreting our data of TN malignancies, as can be seen in Table 1.

The model settings to sustain our scenarios can be synthesized as follows:

Standard configuration:

- Node Layer Architecture: The neural network architecture consists of a single hidden layer with 128 neurons, which was followed by two additional layers with 10 neurons each.
- Epochs: The model was trained for 20 epochs (i.e., 20 complete passes through the entire training dataset).
- Batch Size: During training, the data were divided into batches with each batch containing 32 samples.
- Validation Split: 25% of the data was reserved for validation during training.
- Selected Features: The model used 3 to 7 features, including WOind, Visual pattern, hypoechogenicity, microcalcifications, TIRADS and Bethesda (indexed accordingly), as input.

## 3. Results

The total number of study participants was 148, of whom we included 44 patients with nodular goiter (one patient had two analyzed thyroid nodules) that have fulfilled study inclusion criteria and in which we performed ^99m^Tc-MIBI semi-quantitative thyroid scans (visual image results, visual pattern and ^99m^Tc-MIBI wash-out), and 70 patients were addressed for prospective BRAF collection from FNAC. The remain patients (34 patients) were excluded due to abnormal TSH level or PTH level and also due to hot nodules on ^99m^Tc pertechnetate scans, as seen in Figure 1.

From our total of 44 MIBI cohort patients we obtained 135 images (Table 2). There were 39 female and 5 male patients with a median age of 57 years old. For the BRAF cohort, we included 17 male patients and 53 female patients.

**Table 2:** Characteristics of operated patients with histopathologic results and preoperative details of ^99m^Tc-MIBI processed images and BRAF^V600E^ molecular outcome from both cohorts.

	^99m^Tc-MIBI Images	BRAF Results
Total	135	210
PTC	3	7
MTC	0	2
Follicular adenoma	3	3
Minimally invasive follicular carcinoma	1	1
Oncocytic adenoma/carcinoma	0/0	1/1

**Table:** 

Patients’ Name Initials	Sex	Age	Nodule Size	TIRADS	BETHESDA	BRAF V600E c.1799T > A/BRAFV600Ec.1799_1800TG > AA/BRAFV600K c.1798_1799GT > AA	Visual Pattern	WOind	Histology
AT	F	71	25 mm	5	2	WT/WT/WT	B	−31.18	Follicular adenoma
CM	F	64	27 mm	4	1	-	B	−30.73	Adenomatous goiter
CD	M	62	31 mm	5	1	-	B	−11.36	Papillary microcarcinoma
DE	F	64	26 mm	4	2	-	B	−4.70	Adenomatous goiter
EE	F	55	25 mm	4	1	-	C	−12.5	Adenomatous goiter
PD	F	42	17 mm	4	3	HZ/WT/WT	C	−6.35	Minimally invasive follicular carcinoma
RE	F	58	24 mm	5	2	-	C	−6.66	Follicular adenoma
VA	F	77	43 mm	5	1	-	C	−10.05	Papillary microcarcinoma
BR	F	36	18 mm	4	1	HZ/WT/WT	B	−38.16	Follicular adenoma
CS	M	69	30 mm	4	2	WT/WT/WT	B	−11.36	Papillary microcarcinoma
TL	F	52	15	5	5	MT/WT/WT	-	-	Papillary carcinoma, follicular variant
AO	F	76	40	5	2	HZ/WT/WT	-	-	Oncocytic carcinoma
LG	F	56	10	4	-	X/X/X	-	-	Medullary carcinoma
HM	F	55	13	5	-	X/X/X	-	-	Papillary carcinoma
PR	M	53	30	5	3	X/X/WT	-	-	Oncocytic adenoma
OC	F	22	38	4	-	X/X/X	-	-	Medullary carcinoma
AE	F	67	30	5	5	HZ/WT/WT	-	-	Papillary carcinoma, follicular variant
IV	F	65	40	5	1	HZ/WT/WT	-	-	Papillary microcarcinoma
PE	F	53	40	5	2	X/X/X	-	-	Follicular adenoma
CR	F	58	21	4	2	X/WT/X	-	-	Adenomatous goiter
JE	F	73	50	4	2	MT/WT/WT	-	-	Papillary microcarcinoma, follicular variant
BL	F	42	25	4	2	WT/HZ/WT	-	-	Papillary microcarcinoma

PTC—papillary thyroid carcinoma, MTC—medullary thyroid carcinoma, WT—wild type, MT—mutated, HZ—heterozygote, X—no detection, WOind—wash-out index.

Out of the total number of patients included in the study, only 22 patients managed to obtain the final histopathological result through thyroidectomy: nine PTC patients, one minimally invasive follicular carcinoma, two MTC patients, oneoncocytic carcinoma, oneoncocytic adenoma, four follicular adenomas and four adenomatous goiter patients.

**Figure 4:**
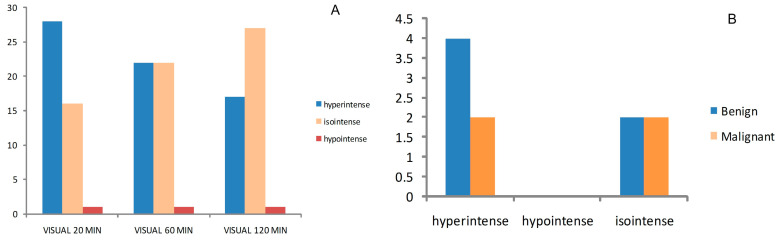
Total thyroid nodules distribution in our MIBI cohort explored with visual ^99m^Tc-MIBI scale (**A**) and the available histopathological result of visual assed thyroidectomized patients (**B**).

MIBI scan results of 44 patients (one patient had two analyzed TN) were interpreted by two experienced nuclear medicine doctors with the following outcomes: ^99m^Tc-MIBI planar visual images (hyperintense, isointense and hypointense) for 20 min, 60 min and 120 min after the injection, as described in the Methods section. We have found that thyroid nodules are more frequently isointense in our study: 27 TN on late images (120 min) were isointense compared to 1 hypointense TN and 17 hyperintense TN. We also found that two hyperintense TN and two isointense TN were malignant out of all of the acquired images (20 min, 60 min, 120 min), as seen in Figure 4.

**Figure 5:**
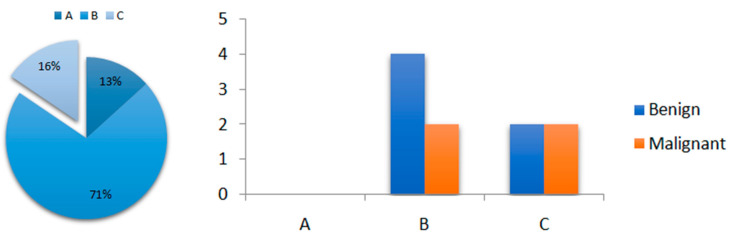
^99m^Tc-MIBI kinetics (pie) and number of patients diagnosed with malignant thyroid nodule from the available histopathological results (columns), depending on ^99m^Tc-MIBI visual pattern: Visual pattern A: reduced uptake in the nodule in the early and late image; Visual pattern B: uptake in the nodule that decreases from early to late image; Visual pattern C: uptake in the nodule that remains unchanged or has further increased on the delayed image [20,21,22].

After conducting the visual assessment of wash-out kinetics of ^99m^Tc-MIBI in our study, pattern A was found in 6 thyroid nodules (13%), pattern B was found in 32 thyroid nodules (71%) and pattern C (16%) was found in 7 nodules (Figure 5).

We compared these kinetics results with the histopathological results, and we found that for pattern C, which is considered suspicious for malignancy, two patients had malignant thyroid nodules but also two patients with pattern C were benign (Figure 5).

**Figure 6:**
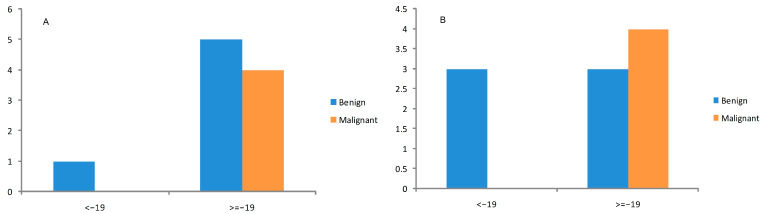
Wash-out index results of our study’s thyroid nodules compared with final histopathological results. (**A**) Wash-out index at 60 min by injection, (**B**) wash-out index at 120 min by injection. The following formula was used according to Campenni et al.:WOind (%) = LR/ER × 100 −100 [20,21,22].

**Figure 7:**
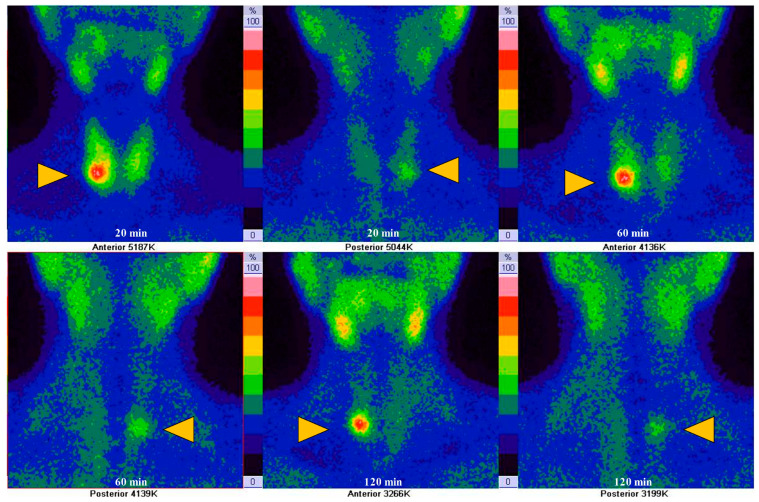
Anterior and posterior images of 42-year-old female with a TN (yellow triangle) presenting: ^99m^Tc-MIBI WOind 60 min = −9.24% and Woind 120 min = −6.35% (low wash-out), Bethesda III in FNAC, positive BRAF mutation and minimally invasive follicular carcinoma on histopathology.

The wash-out index was calculated as outlined in the Methods section, and the cutoff of −19% was used to interpret our study patients’ nodules results. We found a wash-out >−19% (low wash-out) in 8 patients for the 60 min images and for 21 patients at 120 min after the injection A high wash-out index of <−19% was found in 37 patients at 60 min and for 24 patients at 120 min images. The histopathological results were correlated, and we found that thyroid nodules with a wash-out index of >−19% (low wash-out) indeed were malignant in four patients at 60 min and 120 min, as seen in Figure 6 and Figure 7.

For this study, we found a total ^99m^Tc-MIBI imaging sensitivity of 75% and an NPV of 83.82%. Also, for the wash-out index of >−19% (increase possibility of malignancy), NPV was about 90% with an accuracy of 70% for the 120 min late images, as reported in Table 3.

Our molecular results, from the prospectively FNAC collection, became detectable for BRAF mutation in 43 patients (62.31%) from our total of 70 patients. BRAF results were: BRAF^V600E^ c.1799T > A with 28 results of the wild type (WT—negative BRAF mutation) and 9 results of positive BRAF nodules, BRA ^V600E^ c.1799_1800TG > AA with 37 WT results and 1 positive patient, as well as BRAF^V600K^ c.1798_1799GT > AA with 41 WT results and no positive mutation.

**Figure 8:**
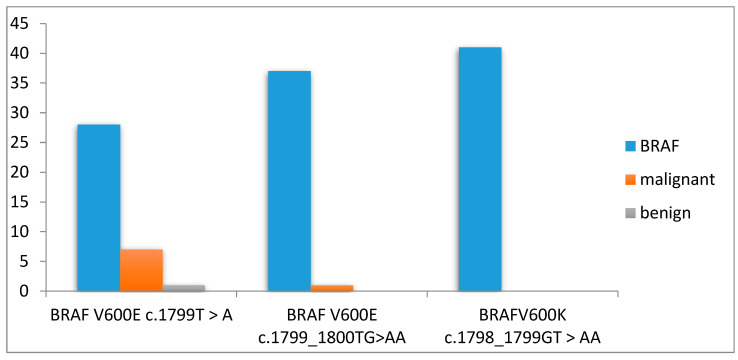
Molecular FNAC—BRAF results from our study, total BRAF detection in thyroid nodules (blue columns)and proportion of malignant TN (orange columns) and benign TN (gray columns) results from the available histopathological results.

We compared our molecular results with the histopathological outcomes, and we found that 7 patients out of a total of 22 patients that were surgically treated (due to the COVID-19 pandemic, only a few patients were able to be surgically treated) had positive BRAF malignant thyroid nodules. Six patients were positive for BRAF^V600E^ c.1799T >A, and one patient was positive for BRAF^V600E^ c.1799_1800TG > AA, as seen in Figure 8. Five patients were confirmed by histopathology to papillary thyroid carcinomas. The other two patients were confirmed with oncocytic carcinoma (one patient) and minimally invasive follicular carcinoma (one patient) and had positive heterozygote (HZ) BRAF^V600E^ c.1799T > A.

**Figure 9:**
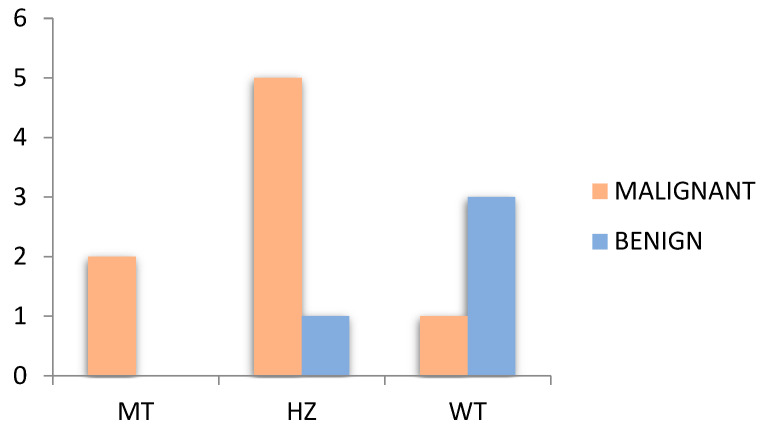
Presentation of benign and malignant cases in our small BRAF cohort who underwent surgery.

We observed that two malignant patients were positive for BRAF^V600E^ MT, five malignant patients were positive for BRAF^V600E^ HZ, and only one patient had benign TN with a positive BRAF^V600E^ HZ mutation (Figure 9). For the wild type (negative BRAF mutation), we found one patient that was diagnosed with malignancy and three patients with benign TN. From the BRAF cohort, all operated BRAF positive patients were diagnosed with papillary and follicular carcinoma.

Our study’s diagnostic performance for molecular findings of BRAF cohort shows us a PPV of 87.50%. We corroborated 70 molecular samples (43 BRAF detectable results) with 22 histopathological findings. Our study specificity for BRAF findings from FNAC was 75% with a sensitivity of 87.50% and accuracy of 83.33% as seen in Table 4.

AI model

In addition to the scintigraphy results and molecular findings, we tried to detect a certain pattern of functional images of malignant thyroid nodules with our AI model, consisting at first of two numerical indicators—WOind taken at different intervals—and a categorical indicator—the visual pattern. Correlated with the information obtained from postoperative pathology reports, we could assess the risk of a malignant TN for the patients that did not have surgery.

Data Augmentation

Although the data provided interesting patterns (obvious even for nonmedical professionals), the number of examples was not enough for machine learning. Also, some examples that seem to indicate a verdict that counters expectations may lead to incorrect patterns. There are the following explanations for the surprising verdict: the pattern only indicates a probability, so a patient can be in a different situation than anticipated, or there is another variable we need to watch. We picked by hand seven examples in order to train the model (four from healthy patients), and most of them were assessed as expected by the medical specialist. For each example, we made a hundred copies, varying the WOind randomly within the [−2.5, 2.5] interval. Corrections were also applied in order not to have a value that is too small or large. This variation leads to more examples to train, and it is small enough in order not to affect the outcome too much.

Some problems were identified regarding the used data or new features. For example, not all the features are useful for the machine learning processing or for identifying a pattern in general; some features are too complex in the original form and required a manual transformation (removing the natural language) by the medical experts into information that can be processed by the machine learning model. Also, some measurements were taken from different patients; therefore, it is not possible to use all the information in one run, and we chose to use only a small group of patients with several features.

We added new features:

- TIRADS—values of 3, 4, and 5; they do not seem to be represented equally;
- Bethesda—similar scale, the value is missing for several patients;
- Hypoechogenicity—0 (low risk)/1;
- Microcalcifications—0 (low risk)/1.The value tends to indicate if the patient is at risk or not; however, we must not rely on it always, as some examples could not follow the observed pattern (but from the existing data of patients who had surgeries, there were no such cases).

We tried the following combinations (WOind and the visual pattern are present in every experiment):

- With TIRADS and Bethesda;
- With hypoechogenicity and microcalcifications;
- With all of the above.

Model

The model architecture is not special considering the processed data are simple. The neural network has four dense layers with a particular configuration of neurons (the numbers were determined by experiments)—128, 10, 10, and 1. Fewer layers brought a smaller score, but complicating the architecture (i.e., too many neurons) also created problems, which was probably because of the data structure or the small number of examples. We carefully chose the number of epochs as 20, as from a certain point (we also tried 50), the model may overfit, and even if the results would be better for a larger number of epochs on training/validation data, the model would lose the ability to generalize. The batch size is 32; we also tried 8, but the overfitting risk was bigger. The proportion represented by validation data is 25%. The data are preprocessed: we brought the WOind measurements into the [−1, 1] range, the visual pattern is coded so it can be in [0, 1], and so on for the other features where necessary.

Metrics and results:

We used accuracy to determine if our model works. We view the initial score as approximately 98% (it can be greater, for example with more epochs, but we need to take into account the overfitting tendency and the fact that the microcalcification is excluded and it might have brought a different verdict), and considering we used a small number of examples to train the model, it has potential. We also created some test examples, and the model indicates the expected outcome most of the time. With the new features, when microcalcification was included, the score was 100%. The other new features (TIRADS, Bethesda) seem to usually bring small improvements because of a few histopathological results in order to compare with non-operated patients, and at the same time, not all our cohort had FNAC with Bethesda results.

- Loss (Training Loss): The loss value represents how well the model’s predictions match the actual target values during training. Lower values indicate better performance. We obtained 0% loss when all features were added (WOind, visual pattern, hypoechogenicity, microcalcification) and 8%-26%when WOind and visual pattern were added alone.
- Accuracy (Training Accuracy): The accuracy value indicates the proportion of correctly predicted samples in the training dataset. We obtained 100% accuracy when all selected features were added (WOind, visual pattern, hypoechogenicity, microcalcification) and 98%-100% accuracy for WOind and visual pattern alone.
- Validation Loss (val_loss): The validation loss measures how well the model generalizes to unseen data (validation set). Similar to training loss, lower values are desirable. We obtained a 0% validation loss when the accuracy was 100% for all selected features and 8%-26%validation loss when WOind and visual pattern were added alone.
- Validation Accuracy (val_accuracy): The validation accuracy reflects the model’s performance on the validation set. It is essential to monitor this metric to avoid overfitting. We obtained a 100% validation accuracy when all features were added and 98%-100%when WOind and visual pattern were added alone.

## 4. Discussion

Our study was a prospective study that combined functional images with FNAC molecular findings in patients that already have surgical indication: nodular size, compressive local effect, and ultrasound characteristics (TIRADS 4/5), which allowed us to add algorithm items in order to surgically treat the patient.

With this prospective study, we found out that ^99m^Tc-MIBI may help in the personalized diagnostic workup of a thyroid nodule, especially where indeterminate FNAC is present, or when Bethesda I/II is confirmed, in a patient with a highly suspicious thyroid nodule. Taking into account the laboratory tests, thyroid US—TIRADS, thyroid scans with pertechnetate, the ^99m^Tc-MIBI functional images and the FNAC—shows an individual approach to the presurgical thyroid algorithm. Prospective molecular findings from FNAC show us that BRAF mutation is an aspect that cannot be neglected in the process of surgical treatment.

We maintained the same wash-out index cutoff (−19%) as presented by Schenke et al., Campenni et al., and Giovanella et al. [20,21,22], and our results are similar with their findings, with an NPV of 90% on late images (120 min) and with an accuracy of 70%. We have brought a novelty into the late images semi-quantitative process, which was not addressed in the previous articles: the inclusion of 60 min images [23], which carries a great risk for malignancy. Our 60 min late images had an NPV of 86.62% with a sensitivity of 80%. However, the comparison between wash-out index at 60 min and 120 min showed us that many nodules were malignant by >−19% WOind at 120 min, which was followed by 60 min. Also, our calculated *p* value of 0.2 for 120 min WOind is statistically insignificant, and we comment on this value by the fact that the group of patients addressed to the surgical intervention was not large enough.

The retention of ^99m^Tc-MIBI uptake in benign thyroid nodules may occur in hyperplasic nodular goiter, macro- and microfollicular adenoma, oncocytic (Hürthle) cell adenoma, or autoimmune or subacute nodular thyroiditis, as reported by Giovanella et al. and Rager et al. [24,25]. These findings are also reinforced by the results of our study: the late accumulation of ^99m^Tc-MIBI radiotracer at 60 min or 120 min revealed many benign thyroid lesions like the ones stated above. Theseshould not be neglected, as these benign variants of thyroid nodules, microfollicular adenoma, and oncocytic (Hürthle) cell adenoma can turn into malignant lesions over the years due to the inherited and microenvironment mutational status, as reported by Correia et al. [26], Fagin et al. and Dralle et al. [27,28]. The expression of proliferative cell nuclear antigen (PCNA) was greater in parathyroid glands with high ^99m^Tc-MIBI scores (hyperintense parathyroid adenoma) compared to those with low scores (hypointense parathyroid adenoma) [29]. For ^99m^Tc-MIBI-positive nodules, it has been proven over time that the expression of proliferative cell nuclear antigen was greater in hyperintense^99m^Tc-MIBI nodules. Those nodules benefited from surgery and proved to be benign, but they still carried a higher risk of progressing into malignant nodules due to PCNA expression. PCNA is a key protein of abnormal cell proliferation. This gene is also expressed in other many tumors: breast, lung, gastric, liver, colon and bladder cancer [30,31,32].

Sometimes, because of extended calcification, it is really difficult to prepare a good smear with enough cellularity, leaving the pathologist to answer the sample as Bethesda II, benign cytology [4]. A negative ^99m^Tc-MIBI scan consistently excluding malignancy with high sensitivity and NPV; a positive ^99m^Tc-MIBI scan, on the other hand, can be observed in both malignant and benign tumors, lowering its specificity and PPV. Lower specificity is related to a non-exclusive metabolic pattern of ^99m^Tc-MIBI distribution also found in multinodular goiter, macro and microfollicular adenoma, Hürthle cell adenoma, or autoimmune nodular thyroiditis, where increased mitochondrial activity could be expected [24]. As a result, a ^99m^Tc-MIBI scan that is positive should be interpreted as “indeterminate”, and for that, the semi-quantitative WOindex is needed in order to conclude a presurgical functional imaging protocol [33].

In our study, we used SPECT images only in inconclusive planar images (only in one patient) because SPECT/SPECT-CT implies higher costs and additional radiation exposuredue to the increase in investigation time, leading to the non-compliance of the other patients. Planar images and ultrasound images are the most cost-efficient, and they are also less radiant, thus being preferred both by patients and medical professionals.

Scintigraphic evaluation was not hindered by TSH target values (<0.5 mUI/L); we conducted the functional images for all normal TSH level patients (TSH normal values chemiluminescent: 0.48–4.17 mUI/L), as conducted in many other studies [34,35].

A semi-quantitative assessment of WOind is employed in only 8% of a recent expert review, while visual and WOind are integrated in the day-by-day routine of also 8%. The adoption of WOind in daily clinical practice is likely limited by the lengthening of the image analysis times and the need for strict standardization of methods. However, while a qualitatively negative ^99m^Tc-MIBI scintigraphy reliably excludes malignancy, many benign follicular proliferations will frequently show isointense or hyperintense^99m^Tc-MIBI uptake and will only be discriminated by a semi-quantitative assessment [35]. In our study, ^99m^Tc-MIBI hyperintense nodules were histological more benign (four patients) then malign (two patients). Visual pattern C in our study, which is considered more prone to be malignant [29], showed us equal results of benignity and malignity (two patients each).

The PPV varies in the published literature between <10% and >61% [36], which is thesame as for our PPV for visual ^99m^Tc-MIBI images that was 11.4% and for WOind at 120 min that was 12.82%. The utility of this value can be relevant for the indication for surgery. Yordanova et al. show that the best PPV was obtained in the interpretation of the tracer uptake trend. If a lesion has an increasing uptake in the late images, the PPV is 32%, and this can justify surgery [36].

Comparing ^99m^Tc-MIBI scanning and FNAC, ^99m^Tc-MIBI has been reported to have a very high NPV ranging from 88 to 100%, with a mean of 97% [37,38], as seen in our study results also.We found a ^99m^Tc-MIBI visual images NPV of 88.6% and for WOind at 120 min a NPV of 90.06%. Furthermore, ^99m^Tc-MIBI scanning for the evaluation of TN may lead to considerable cost savings [39] while not significantly affecting outcomes such as life expectancy.

Another cost-effective way is to repeat FNAC: some studies show that 38.89% TN were malign among the patients who received thyroidectomy after the first FNA, and it increased to 47.50% and 69.23% in the patients with repeated FNAC two or three times [40]. The disadvantages of repeating FNAC are the following: reluctance of the patients to be repeatedly exposed to an invasive procedure, the expenditure of material resources, and the prospect that the final result will possibly be the same as the initial one. The advantages of repeating the FNAC consist of obtaining a valid result in the cases of Bethesda I and also selecting patients for surgical intervention when repeating FNAC brings a Bethesda IV, V result.

For our study, we chose to explore the thyroid nodule through a single puncture from a cytological and molecular point of view at the same time. It brought us the advantage of a much easier acceptance by the patient compared to the repuncture proposal, taking into account the COVID-19 pandemic period when patients avoided exposure to the medical system.

We found a low specificity value for ^99m^Tc-MIBI late images and the^99m^Tc-MIBI wash-out index at 60 min in our study due to the limited number of patients who were referred for surgical intervention. These results need reconfirmation on a much larger group of patients. We also found low specificity (50%) and sensitivity (57.14%) for ^99m^Tc-MIBI wash-out index at 120 min, which is in line with many other literature results [36,41,42,43,44,45] as well as due to the low number of operated patients in the COVID-19 pandemic period, as thyroidectomy was not at all an emergency.

The charge for BRAF mutation analysis is effectively low, with a reported cost for our country (Romania) of 90.54 EUR/test, respectively. Considering their good diagnostic performance as rule-in tests, they could represent a suitable potential screening test for characterizing thyroid nodules with indeterminate cytology. However, BRAF mutation analysis is loaded by a rather low sensitivity, which in our study was 87.50%. We calculated a *p* value of 0.0667, which was interpreted as a statistically insignificant value and also justified by the fact that the thyroidectomy group in the BRAF cohort was also small. It is expressed in a fraction of papillary thyroid cancer (PTC) and in anaplastic thyroid cancer arising from PTC as well as in the follicular variant of PTC (FVPTC), but not in follicular thyroid cancer (FTC) [46].

The cost for FDG-PET/CT in Europe is approximately EUR1.039,80 [47]. However, it should be emphasized that this technique requires a specialized division and a trained team of experts. In addition, this exam exposes the patients to potentially damaging radiation. In this regard, it has been calculated by the International Atomic Energy Agency (see IAEA, safety reports series no. 58, 2008) that the total effective dose for the whole body FDG–PET/CT averages 25 mSv with 8 mSv due to PET and 7–30 mSv due to CT scan elements and final diagnostic scan [48]. Even if the dose can be lowered by examining only the neck, this method appears not suitable as a screening method for thyroid nodules population with indeterminate cytology [46] compared to a^99m^Tc-MIBI scan that use a lot less radiation and costs less as well. The estimated cost per patient for a^99m^Tc-MIBI scan in Romania is EUR120.73.

Considering BRAF a very good rule in tests and also an economic molecular test, we prospectively collected FNAC wash-out precipitation specimens with the result of 31.81% malignancies, meaning seven true positive nodules from 22 operated patients. The difference between molecular results from biopsy and from FNAC wash-out was studied by Cui et al., and the results showed that 74.7% of patients with PTC carried a BRAF^V600E^ mutation in the FNAC biopsy formalin-fixed paraffin-embedded (FFPE) specimens, while the rate detected in the FNAC wash-out precipitation specimens was 54.4% [49]. Considering FNAC wash-out less invasive than biopsy, this technique will be more and more used, especially since BRAF alterations are significantly more common in PTCs with thyroid capsule invasion than in tumors without thyroid capsule invasion [50].

BRAF^V600E^ genetic testing had a huge impact on the overall quality of life and healthcare costs of patients by reducing the number of unnecessary diagnostic thyroid surgeries, including nonfunctional, minimal thyroid adenoma, nodular goiter, and thyroid cyst [51].

A retrospective study including 12,392 patients who had FNAC with or without BRAF^V600E^ genetic testing reflects the application value of BRAF^V600E^ genetic testing for the identification of benign and malignant thyroid nodules before surgery, and they found that the addition of BRAF^V600E^ genetic testing benefited only Bethesda III and V nodules [52]. We selected BRAF samples prospectively according to the TIRADS score, because we would have obtained a small cohort for Bethesda III, IV. We also want to include Bethesda I patients which would have a risk of malignancy of 5–20% [4].

The Bethesda results (49 results) for the collected BRAF samples were as follows: from 15 patients with Bethesda I, one patient was diagnosed with papillary microcarcinoma (6.66%);of the 27 patients with Bethesda II, 3 patients were diagnosed with papillary carcinoma (11.11%);of the 5 patients with Bethesda III,1 (20%) was confirmed as havingminimally invasive follicular carcinoma;there were no patients with Bethesda IV and VI, and 2 patients with Bethesda V were confirmed as having the papillary thyroid carcinoma follicular variant.

From our histopathological examinations, we found out that one patient had benign results (one—follicular adenoma), although BRAF^V600E^ in FNAC was positive. Surgically proven benign cases of false-positive BRAF^V600E^ mutation have been documented in the literature [53,54,55].The real false-positive mutation rate of BRAF^V600E^ testing methods in FNAC specimens might be underestimated; in our study, it was 4.54%. We also found that one patient with PTC had no BRAF mutation at all, which can put us in front of a false negative result of BRAF in FNAC, which is in accordance withZhao CK et al.with 7.1% of false negative BRAF results [54].

With cytological diagnosis alone, the sensitivity and specificity for the diagnosis of PTC was 85.65% and 89.01%, respectively, for a cohort of 516 patients. With the BRAF^V600E^ mutation alone, the sensitivity and specificity for the diagnosis of PTC were 76.71% and 100.0%, respectively. Combining the BRAF^V600E^ mutation with cytological diagnoses increased the sensitivity to 96.62% but decreased the specificity to 88.03% [56].

Molecular assay on the residual FNAC biopsies would be cost-effective in identifying well-characterized histological variations because it reduces the cost and burden to patients by sparing them from a repeated thyroid FNAC procedure, while most other tests require an additional FNAC sample or surgical specimens [57]. BRAF^V600E^ is the most common alternative in cytologically indeterminate thyroid nodules, which is followed by RAS mutations and RET/PTC fusions [7].

The diagnostic role of BRAF^V600E^ assays in thyroid nodules has been fully appreciated due to high incidence, but next-gene sequencing (NGS) panels based on multiple genes and multiple detection types (such as Thyroseq V3, Afirma GEC) provides higher precision diagnostic performance for all common types of thyroid cancer and can simultaneously consider higher sensitivity, specificity, and accuracy but with a higher cost: Afirma GEC—USD 4875 =EUR 4494.02 or the tNGS panel—USD 3200 = EUR 2949.92 [58,59,60,61,62].

Our study has limitations. We had a relatively small cohort for histopathological results, and not all patients could be investigated by ^99m^Tc-MIBI scan after BRAF collection due to thyroid surgery appointment or because the patients disagreed with the 2-day scintigraphy protocol (day one for pertechnetate and day two for ^99m^Tc-MIBI), and they refused toreturn to the hospital during the COVID-19 pandemic, which is why we have two separate ^99m^Tc-MIBI and BRAF cohorts. Due to the COVID-19 pandemic, the number of patients with possible goiter addressability was smaller, and also patients were referred to thyroid surgery (compared to previous years).

The present results, although obtained from a relatively small cohort, contribute to the literature by providing a semi-quantitative functional assessment in eastern Europe. Our semi-quantitative determination requires validation on a much larger scale, which in the future could have the benefit of being automatically calculated by an AI learning system.

The strengths of this study are that we used BRAF molecular samples from FNAC wash-out, patients benefiting from only one needle puncture, and semi-quantitative calculus for ^99m^Tc-MIBI wash-out at 120 min and also at 60 min, which is an efficient and economic predictor for malignancy. We also developed an AI model which could predict if a TN is malignant or not by introducing ^99m^Tc-MIBI WOind and visual pattern analysis, an ultrasound TIRADS score with specific characteristics as microcalcifications and thehypoechogenicity and Bethesda results of FNAC.

Our AI example model generated 100% accuracy of TN characteristics and concluded that patients with WOind of >−19% with visual pattern C, microcalcifications and hypoechogenicity on US examination had an increased possibility of malignancy. We obtained 0% for training loss when all features were added (WOind, visual pattern, hypoechogenicity, microcalcification), and 8%-26% when WOind and visual pattern were added alone. The accuracy was 100% when all of the selected features were added (WOind, visual pattern, hypoechogenicity, microcalcification) and 98%-100% for WOind and visual pattern alone. These results confirm to us that future work for an AI model must include a larger database of US characteristics and scintigraphy scans. Also, more variables in the machine learning model and trying different machine learning configurations (layers, epochs, alter the weights of particular features so they influence the model more etc.) may be required.

## 5. Conclusions

This study highlights the importance of a complete preoperative algorithm through investigations that are costeffective and practical in detecting malignancy. The combination of thyroid ultrasound (TIRADS) with functional (^99m^Tc-MIBI) and molecular investigations (BRAF) brings additional details regarding a positive preoperative diagnosis and surgical indication while avoiding unnecessary surgery for patients who need further monitoring. Due to the economic limitations of the health system and implicit limitations of the patients, we established a personalized algorithm for thyroid nodules that we plan to extend in routine practice. Our results concluded that a positive ^99m^Tc-MIBI scan combined with positive BRAF results could recommend patients for surgery, receiving confirmation for some of the histopathology results in our cohort. We aim to expand the patients group in order to outline a sufficient database for solid statistics. We have also demonstrated that a reliable AI model can be generated to improve the accuracy of the TN presurgical diagnosis, assuring ourselves that the model is trained by the medical practitioner together with the IT specialist to understand the AI parameters introduced by both groups, thus avoiding the medical error generated only by artificial intelligence.

## Figures and Tables

**Table 1:** Summary of the component functions of the proposed AI model for our MIBI cohort.

Component Function	Commentary
Function	*augment (data, repetition, coefficient)*
Purpose	This function generates augmented data by adding random perturbations to the input data.
Input Parameters	Data: a tuple containing input features and corresponding labels. Repetition: an integer specifying how many times to repeat the augmentation process. Coefficient: a numerical value controlling the magnitude of perturbations.
Steps	For each repetition, iterate through each data point in the input features. Perturb the first two values of each data point using the edit function (explained below). Append the modified input features and corresponding labels to separate lists. Return the original input features concatenated with the augmented input features and the corresponding labels.
Context	The function is likely used for data augmentation in machine learning tasks.
Function	*edit (value, coefficient)*
Purpose	This function introduces random noise to a given value.
Input Parameters	Value: a numerical value. Coefficient: a numerical value controlling the range of perturbations.
Steps	Add a random value sampled from a uniform distribution between -coefficient/40 and coefficient/40 to the input value. Ensure the result is bounded between −1 and 1 using min and max.
Context	Used within *augment* to perturb individual feature values.
Function	*reorder (data)*
Purpose	This function shuffles the order of data points.
Input Parameters	Data: a tuple containing input features and corresponding labels.
Steps	Create a list of indices corresponding to the data points. Shuffle the indices randomly. Return the input features and labels in the shuffled order.
Context	Useful for introducing randomness during training or validation.
Function	*read_from_csv (path)*
Purpose	This function reads data from a CSV file.
Input Parameters	Path: a string specifying the path to the CSV file.
Steps	Open the database as a CSV file. Read each line and process it using a process function. Append the processed input features and labels to separate lists. Return the input features and labels.
Context	Used for loading data from external files (CSV format).

**Table 3:** Diagnostic performance of visual ^99m^Tc-MIBI imaging and ^99m^Tc-MIBI wash-out index in our study.

^99m^Tc-MIBI	Sensitivity	Specificity	Accuracy	Prevalence	PPV	NPV	*p* Value
Visual pattern	33%	66.67%	60%	11.4%	11.4%	88.6%	1.00
^99m^Tc-MIBI late images	75%	16.67%	40%	11.4%	10.38%	83.82%	1.00
Wash-out cutoff—19% (60 min)	80%	16.67%	50%	11.4%	11%	86.62%	1.00
Wash-out cutoff—19% (120 min)	57.14%	50%	70%	11.4%	12.82%	90.06%	0.20

PPV—positive predictive value, NPV—negative predictive value.

**Table 4:** Diagnostic performance of BRAF molecular findings in FNAC in our study patients.

Sensitivity	Accuracy	Specificity	PPV	NPV	*p* Value
87.50%	83.33%	75%	87.50%	75.00%	0.0667

## Data Availability

The raw data supporting the conclusions of this article will be made available by the authors on request.
